# Supplementary figures and images for: Small proline-rich proteins (SPRRs) are epidermally produced antimicrobial proteins that defend the cutaneous barrier by direct bacterial membrane disruption
Source: eLife. 2022 Mar 2;11:e76729. doi: 10.7554/eLife.76729 (PMC8912919; doi:10.7554/eLife.76729)

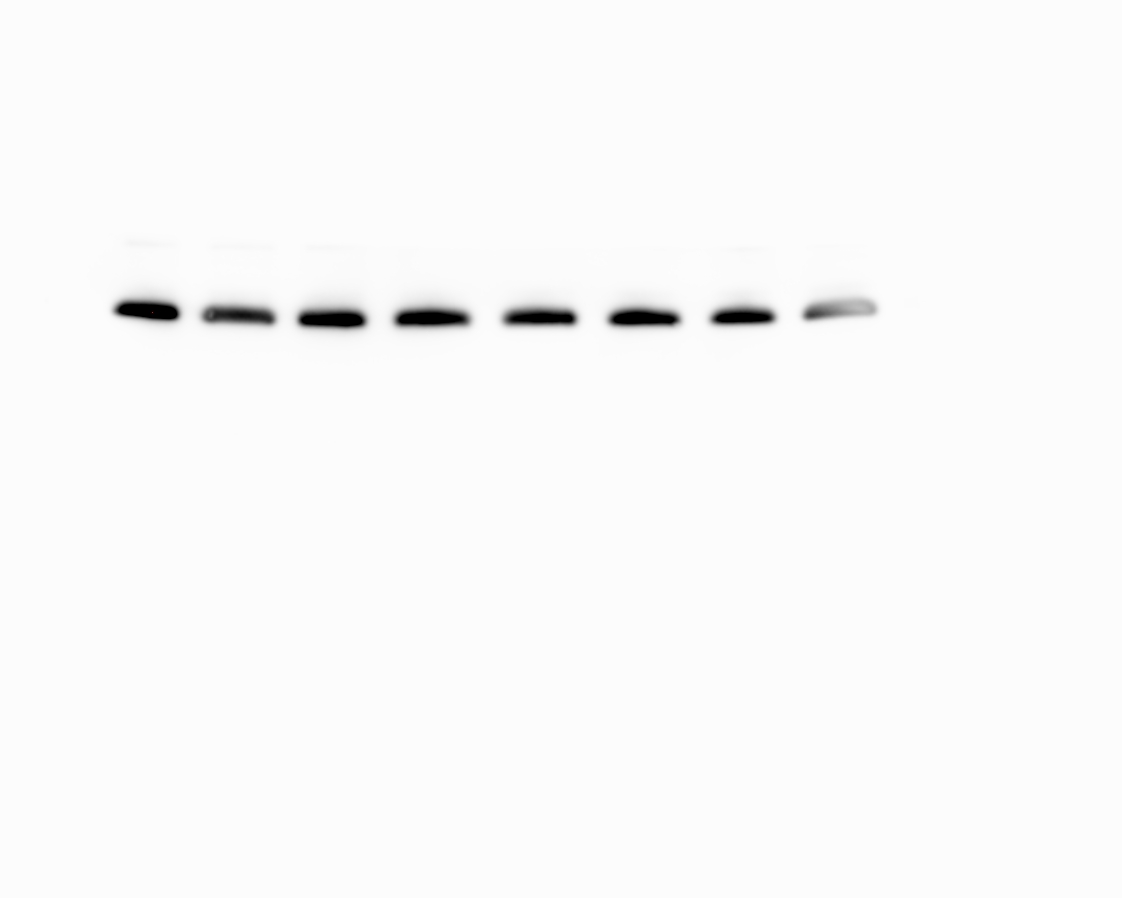

Supplement: Figure 1—source data 1. [file elife-76729-fig1-data1.zip › Figure 1-source data/Figur1GAPDHChenlu 2019-02-13 17h05m30s(Chemiluminescence) - Copy.jpg]

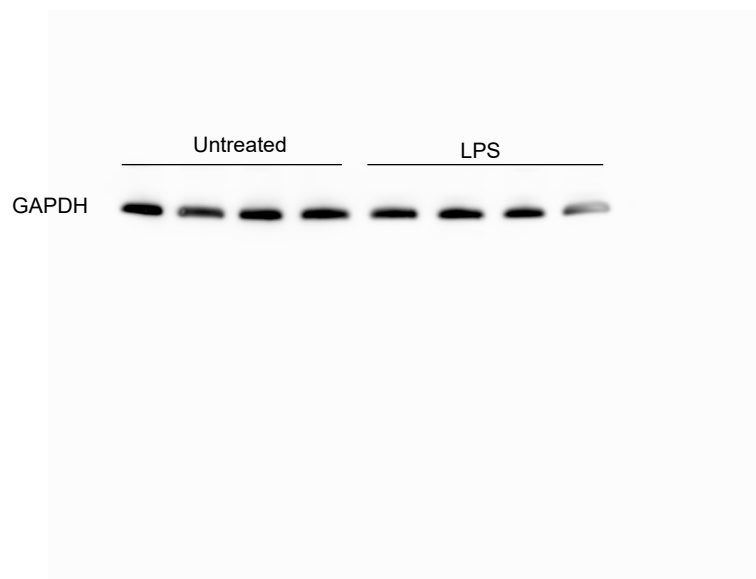

Supplement: Figure 1—source data 1. [file elife-76729-fig1-data1.zip › Figure 1-source data/Figur1GAPDHChenlu 2019-02-13 17h05m30s(Chemiluminescence) - Copy.pdf]

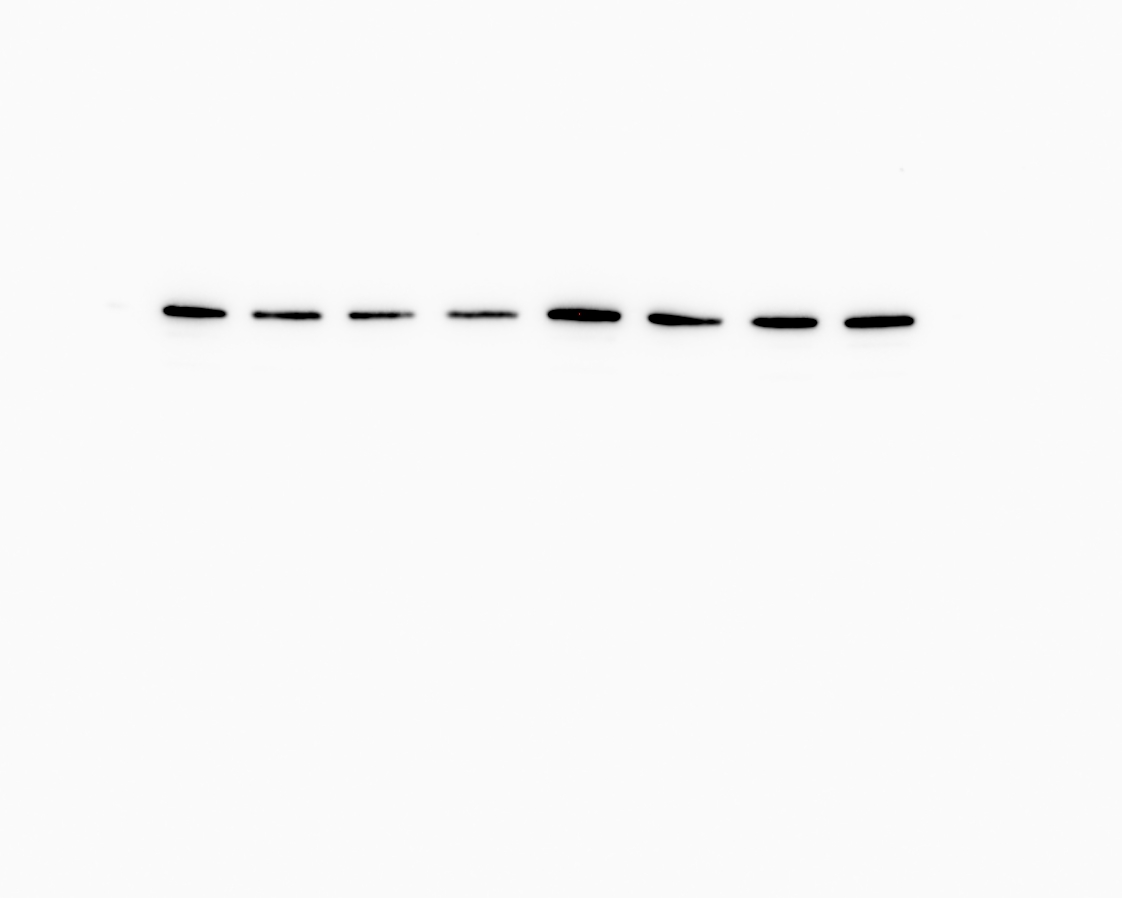

Supplement: Figure 1—source data 1. [file elife-76729-fig1-data1.zip › Figure 1-source data/sprr1bChenlu 2019-02-13 17h10m17s(Chemiluminescence) - Copy.jpg]

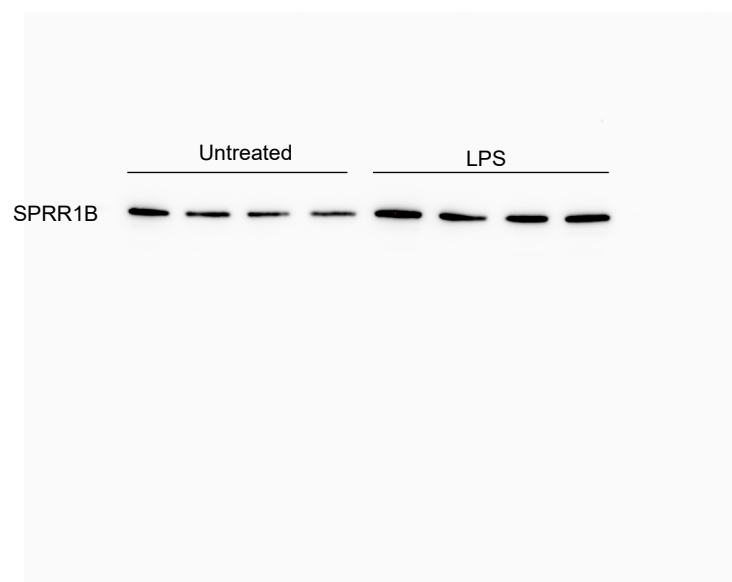

Supplement: Figure 1—source data 1. [file elife-76729-fig1-data1.zip › Figure 1-source data/sprr1bChenlu 2019-02-13 17h10m17s(Chemiluminescence) - Copy.pdf]

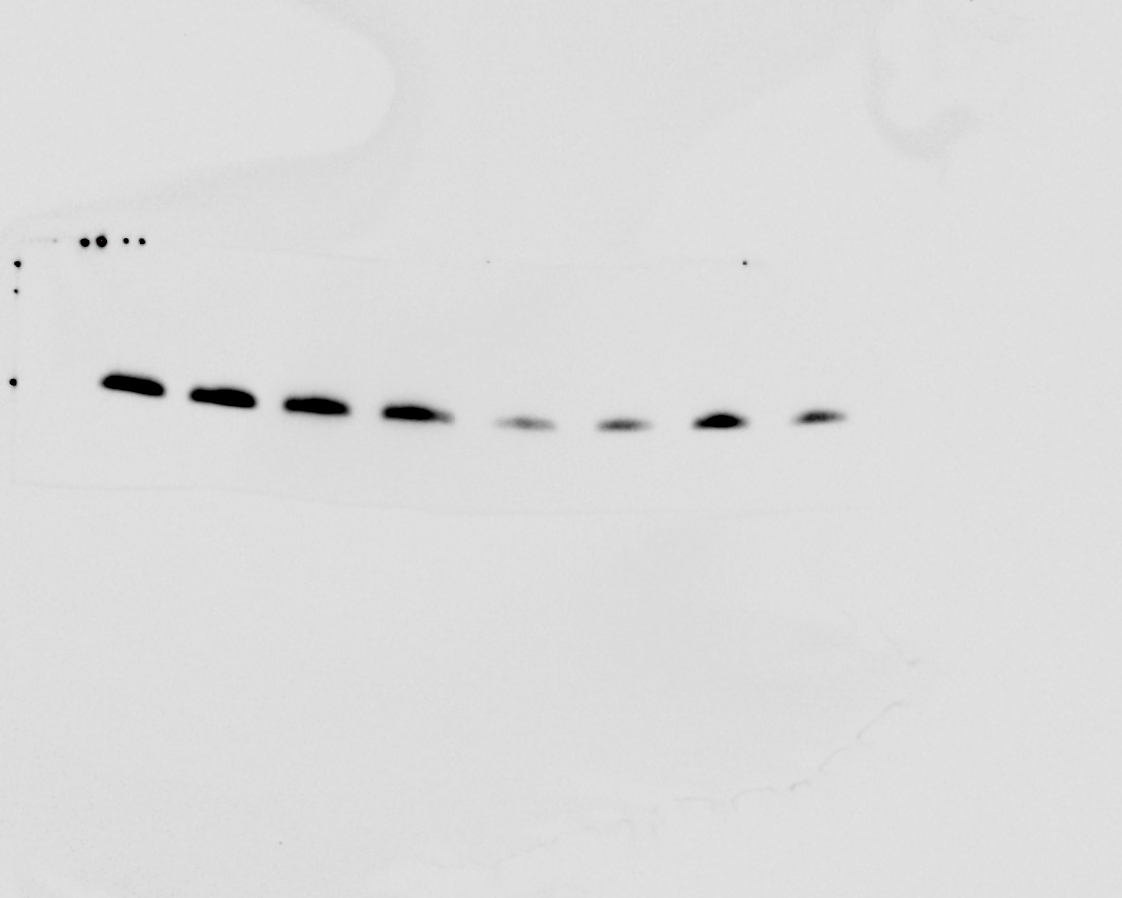

Supplement: Figure 1—source data 1. [file elife-76729-fig1-data1.zip › Figure 1-source data/SPRR2aChenlu 2019-02-13 16h59m29s(Chemiluminescence) - Copy.jpg]

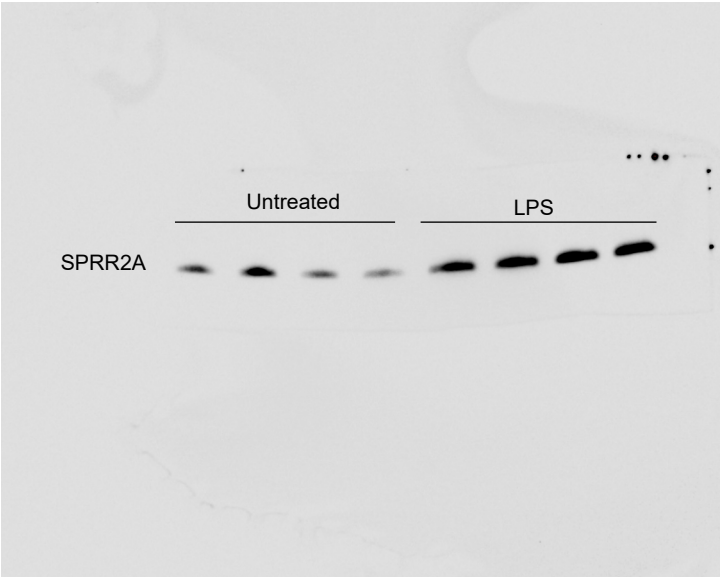

Supplement: Figure 1—source data 1. [file elife-76729-fig1-data1.zip › Figure 1-source data/SPRR2aChenlu 2019-02-13 16h59m29s(Chemiluminescence) - Copy.pdf]

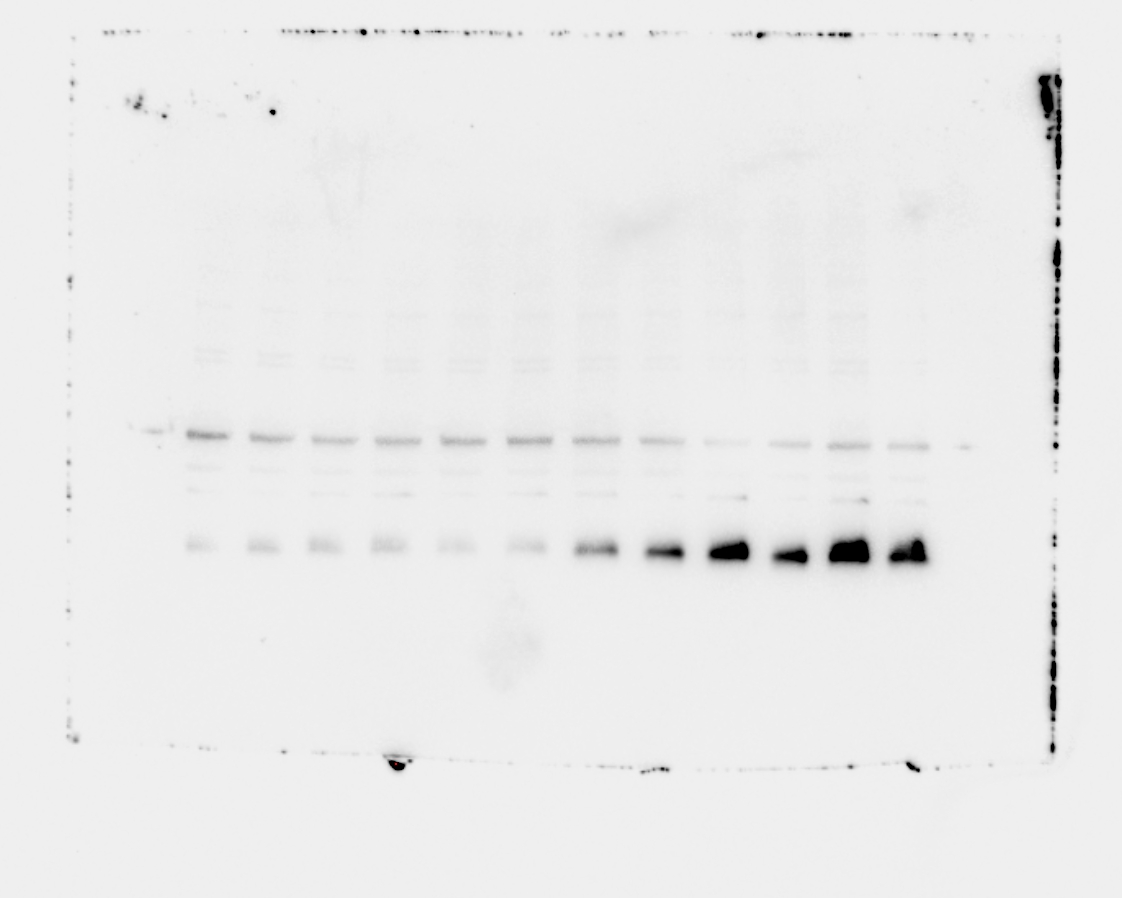

Supplement: Figure 2—figure supplement 2—source data 1. [file elife-76729-fig2-figsupp2-data1.zip › Figure 2-figure supplement 2-source data/Fig2_sup2_sourceChenlu 2019-03-19 18h13m16s(Chemiluminescence).jpg]

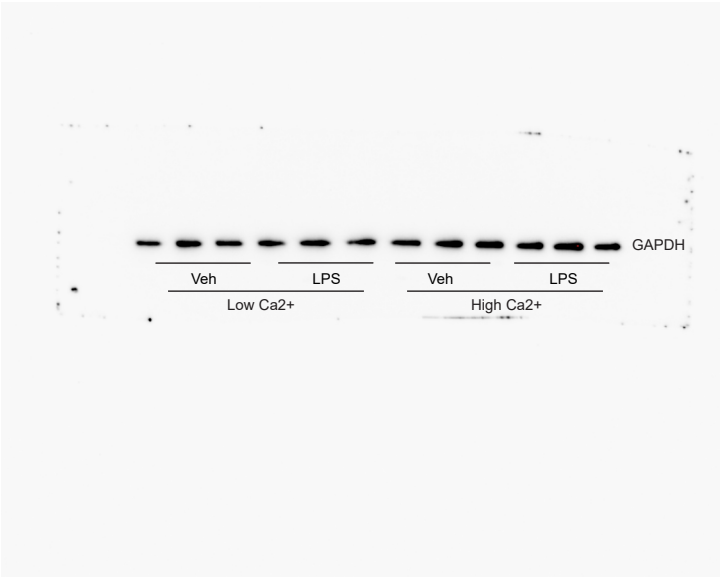

Supplement: Figure 2—figure supplement 2—source data 1. [file elife-76729-fig2-figsupp2-data1.zip › Figure 2-figure supplement 2-source data/Fig2_sup2_sourceGapdh(Chemiluminescence).pdf]

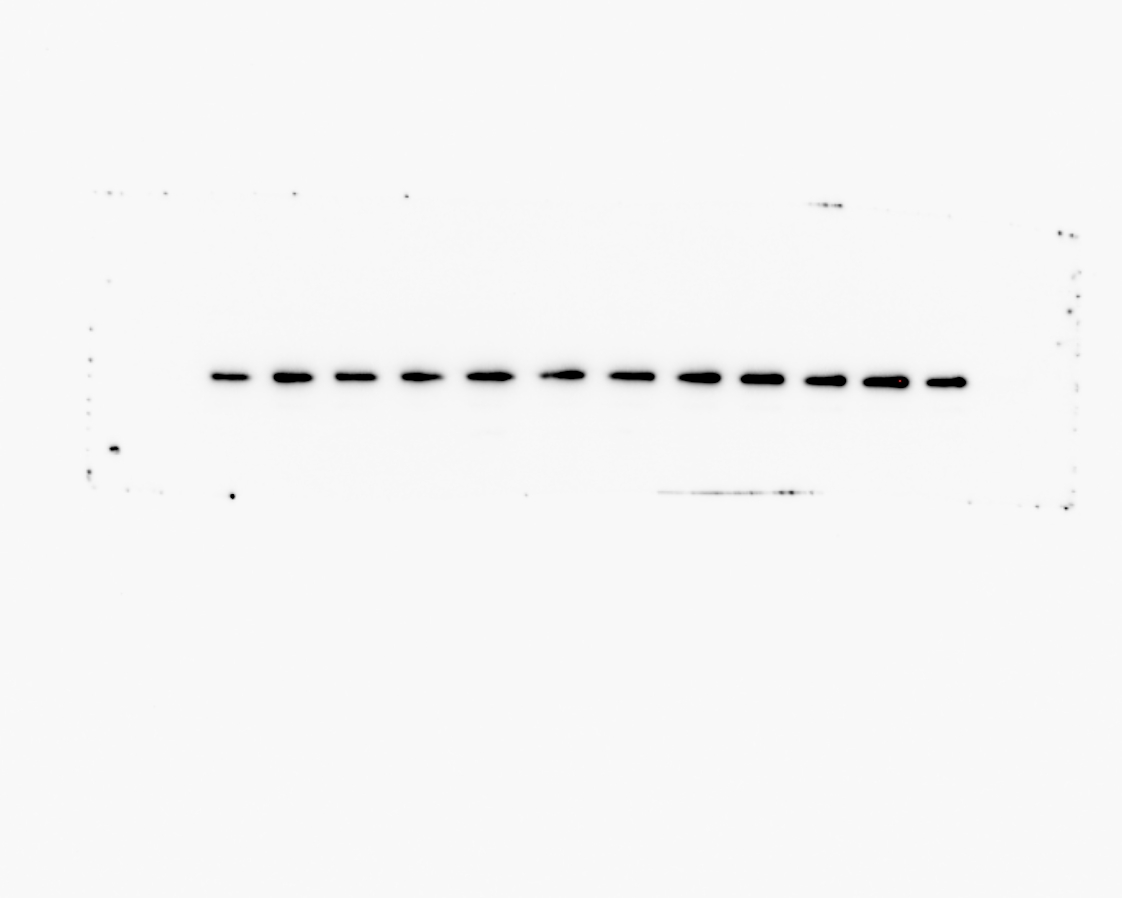

Supplement: Figure 2—figure supplement 2—source data 1. [file elife-76729-fig2-figsupp2-data1.zip › Figure 2-figure supplement 2-source data/Fig2_sup2_sourceGapdhChenlu 2019-03-19 18h06m23s(Chemiluminescence).jpg]

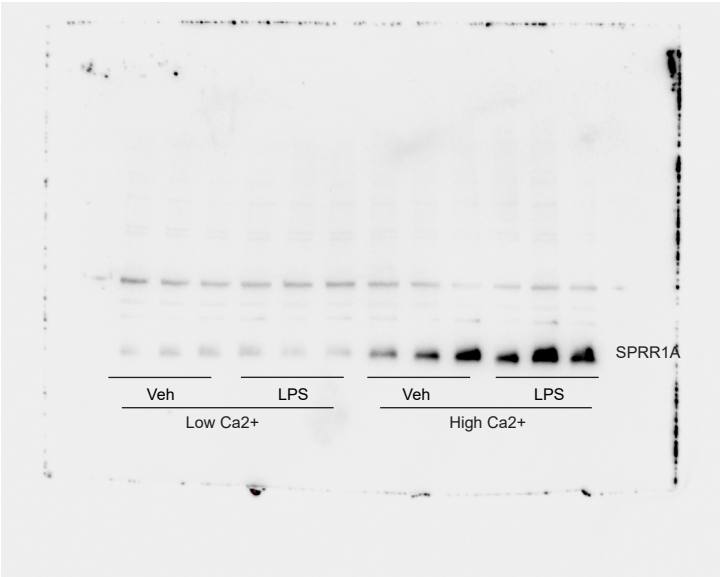

Supplement: Figure 2—figure supplement 2—source data 1. [file elife-76729-fig2-figsupp2-data1.zip › Figure 2-figure supplement 2-source data/Fig2_sup2_sourceSPRR1A.pdf]

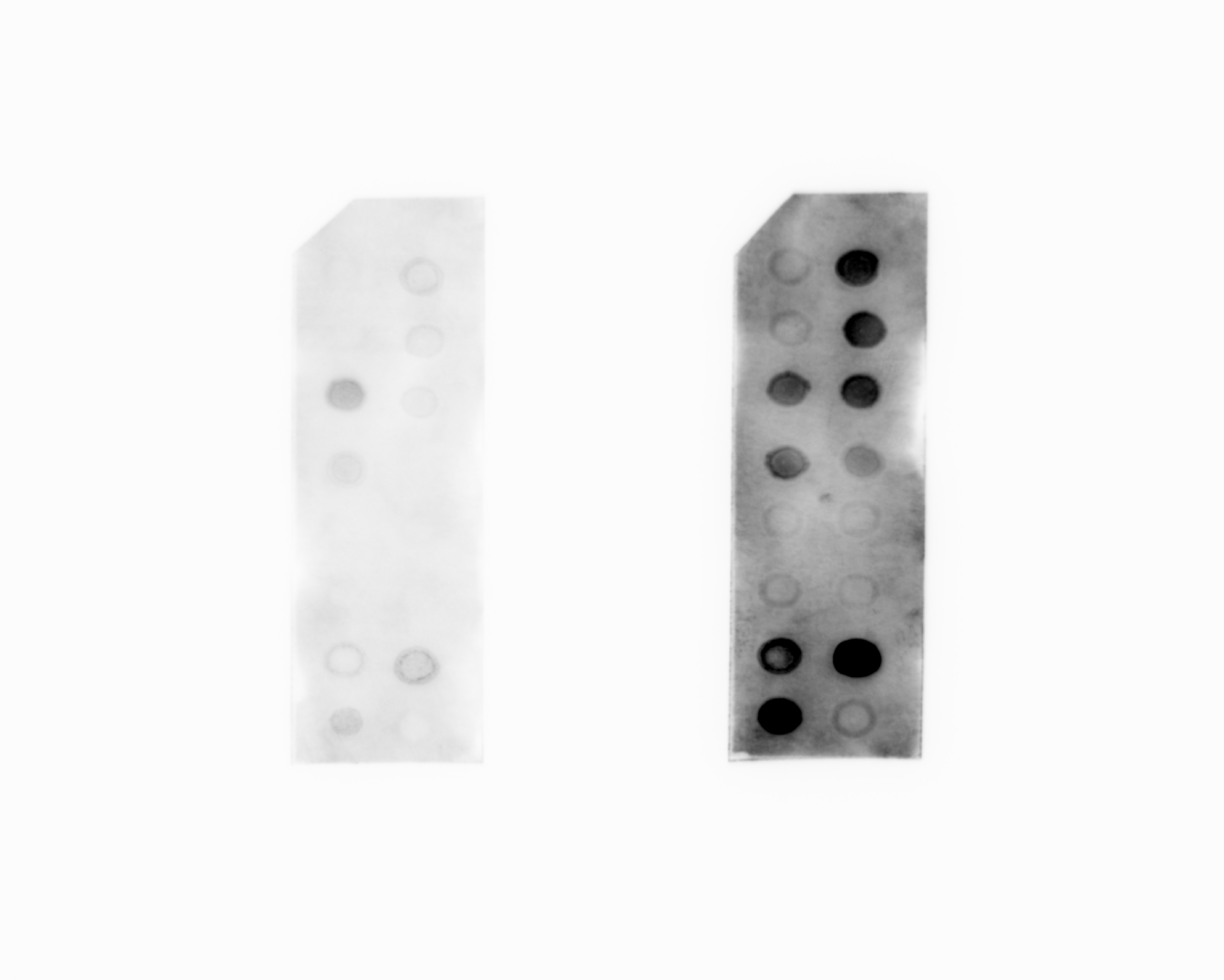

Supplement: Figure 3—source data 1. [file elife-76729-fig3-data1.zip › Figure 3-source data/2021-02-09 15hr 13min 02sec_[right]]hSPRR1B.tif]

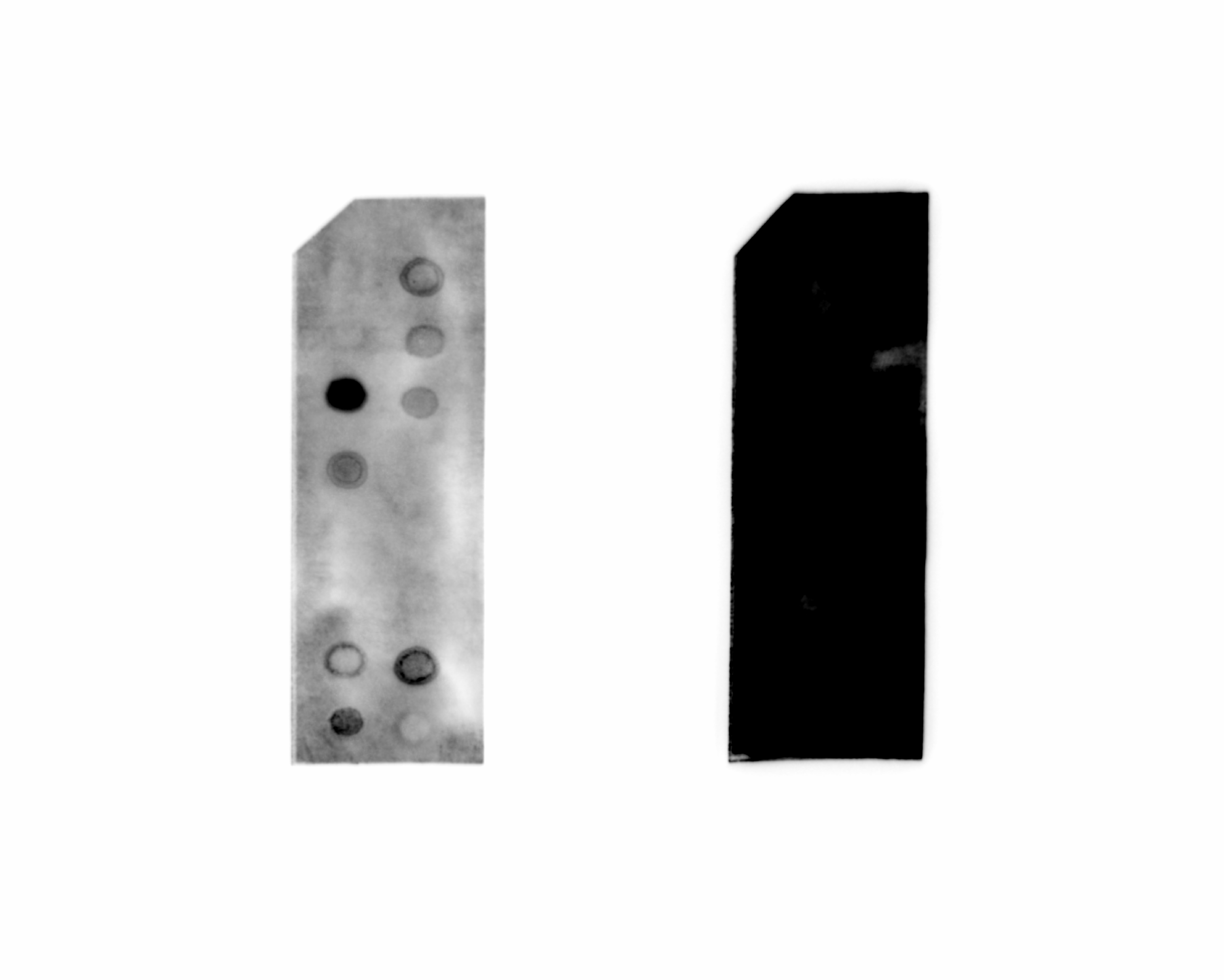

Supplement: Figure 3—source data 1. [file elife-76729-fig3-data1.zip › Figure 3-source data/2021-02-09 15hr 15min 02sec_[left]mSPRR1A.tif]

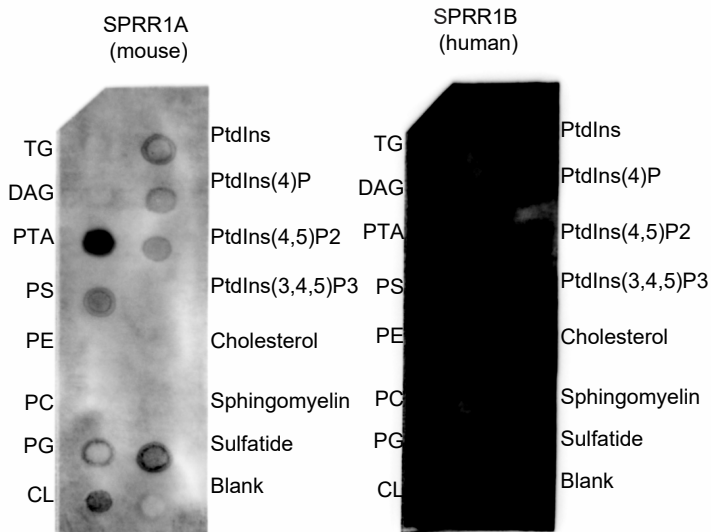

Supplement: Figure 3—source data 1. [file elife-76729-fig3-data1.zip › Figure 3-source data/longexposure.pdf]

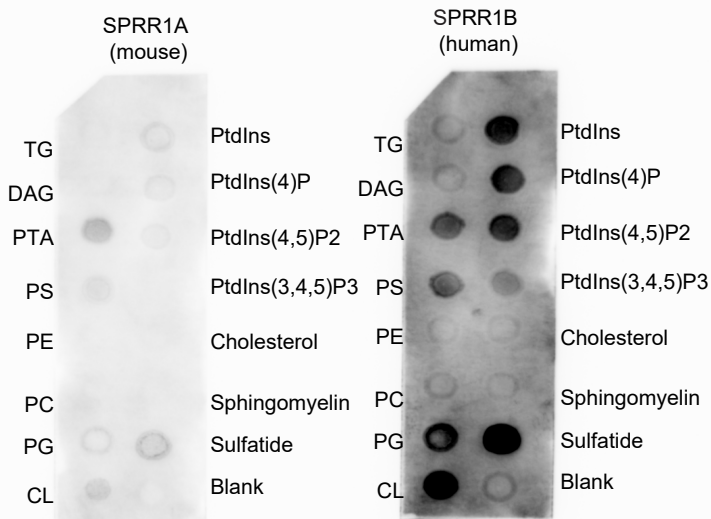

Supplement: Figure 3—source data 1. [file elife-76729-fig3-data1.zip › Figure 3-source data/shortexposure.pdf]

# SPRR1A (mouse)

250 -  
150 -  
100 -  
75 -  
50 -  
37 -  
25 -  
20 -  
15 -  
10 -  
(kDa)

9 10 11 12 13 14 15 16 17

Elution volume (mL)

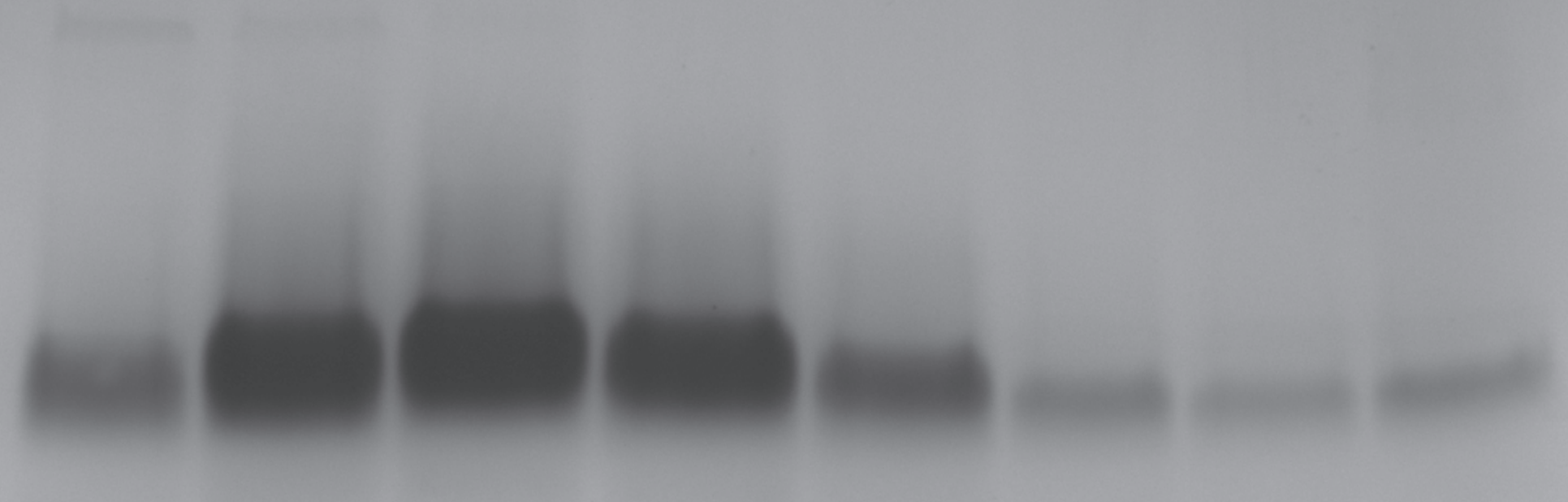

Supplement: Figure 3—figure supplement 1—source data 1. [file elife-76729-fig3-figsupp1-data1.zip › Figure3-figuresupplement1-Source Data/20180807-mSprr1a_SD75.pdf]

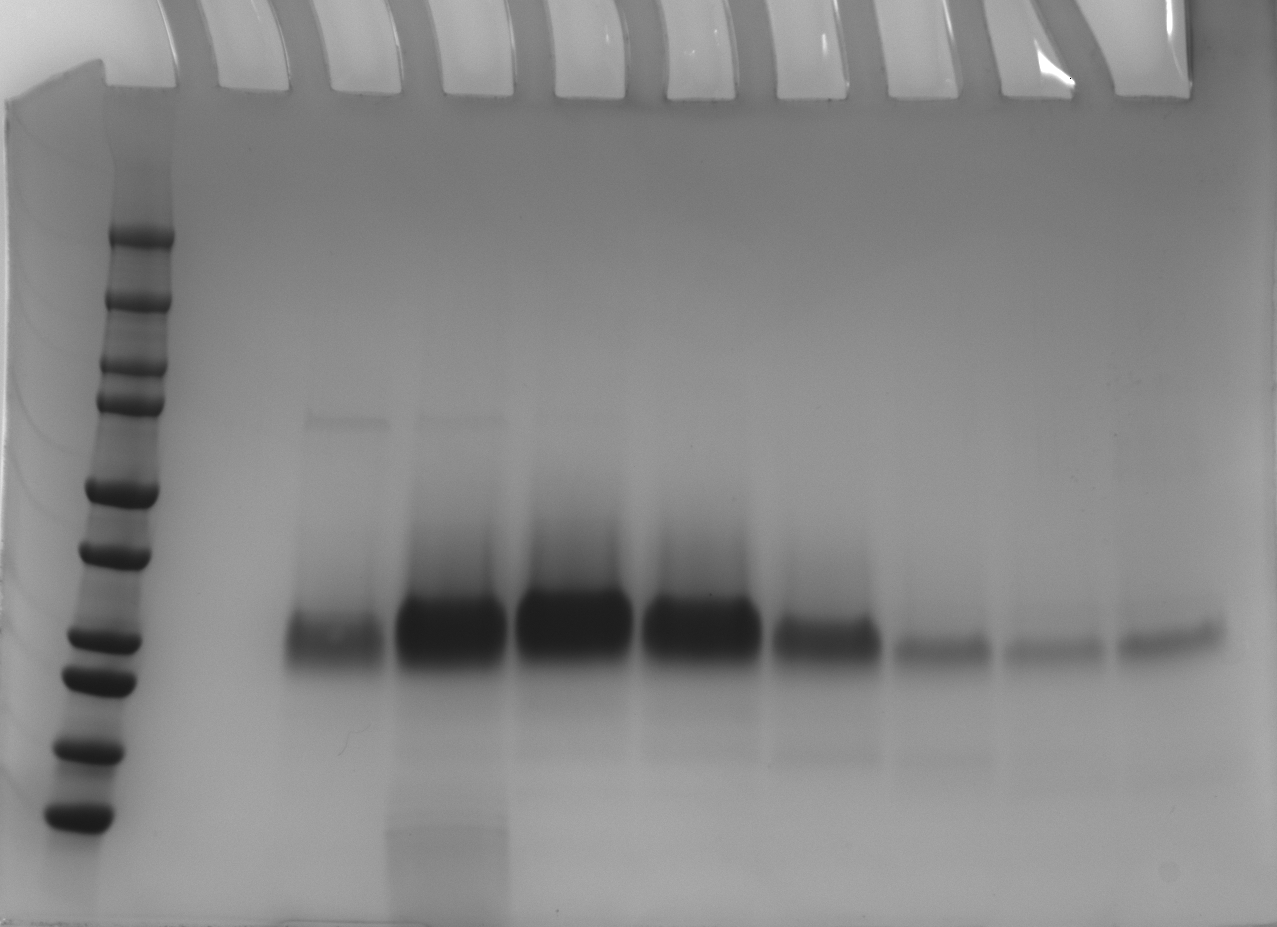

Supplement: Figure 3—figure supplement 1—source data 1. [file elife-76729-fig3-figsupp1-data1.zip › Figure3-figuresupplement1-Source Data/20180807-mSprr1a_SD75.tif]

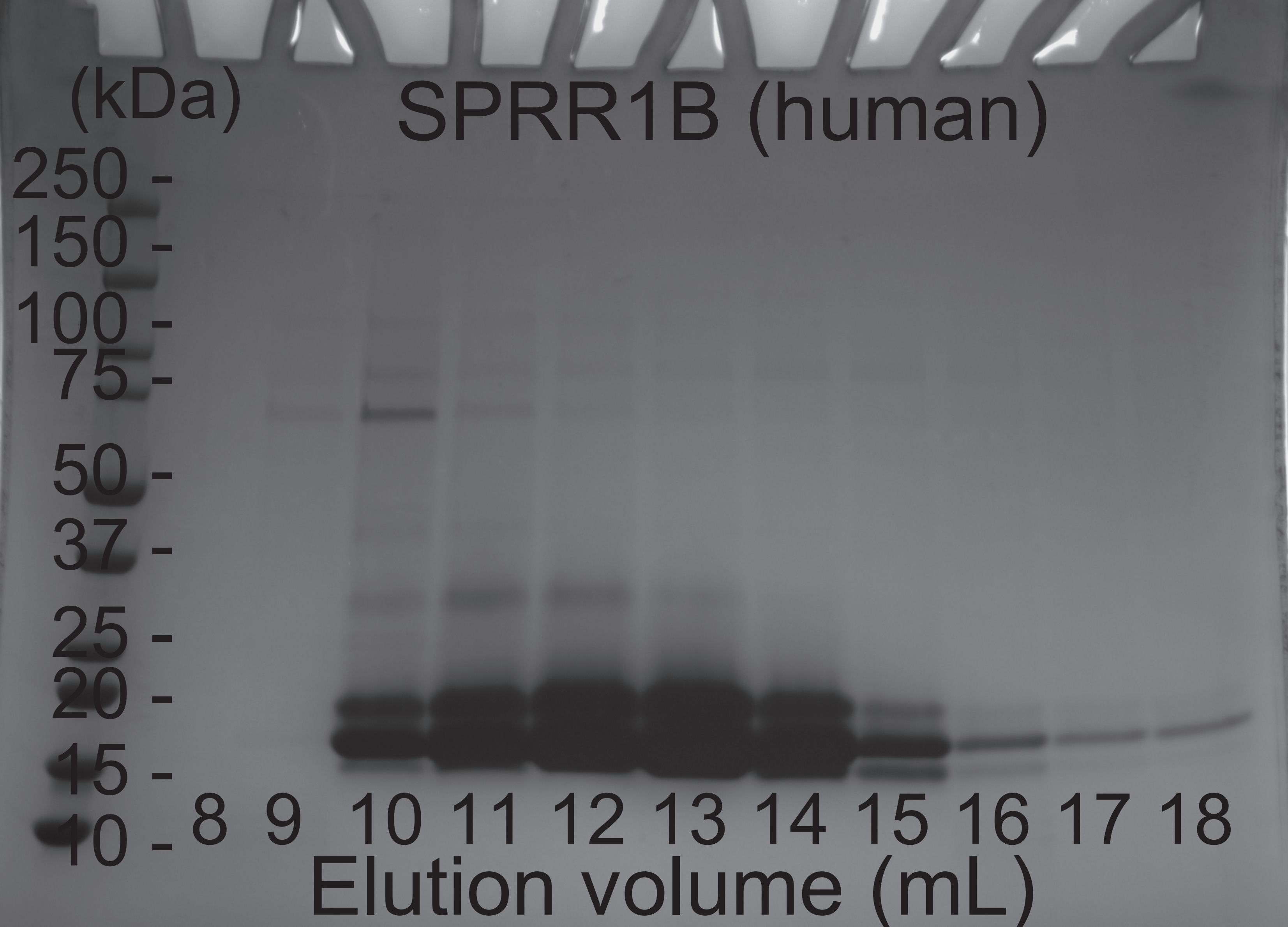

Supplement: Figure 3—figure supplement 1—source data 1. [file elife-76729-fig3-figsupp1-data1.zip › Figure3-figuresupplement1-Source Data/20180920-hSPRR1B-SD75.pdf]

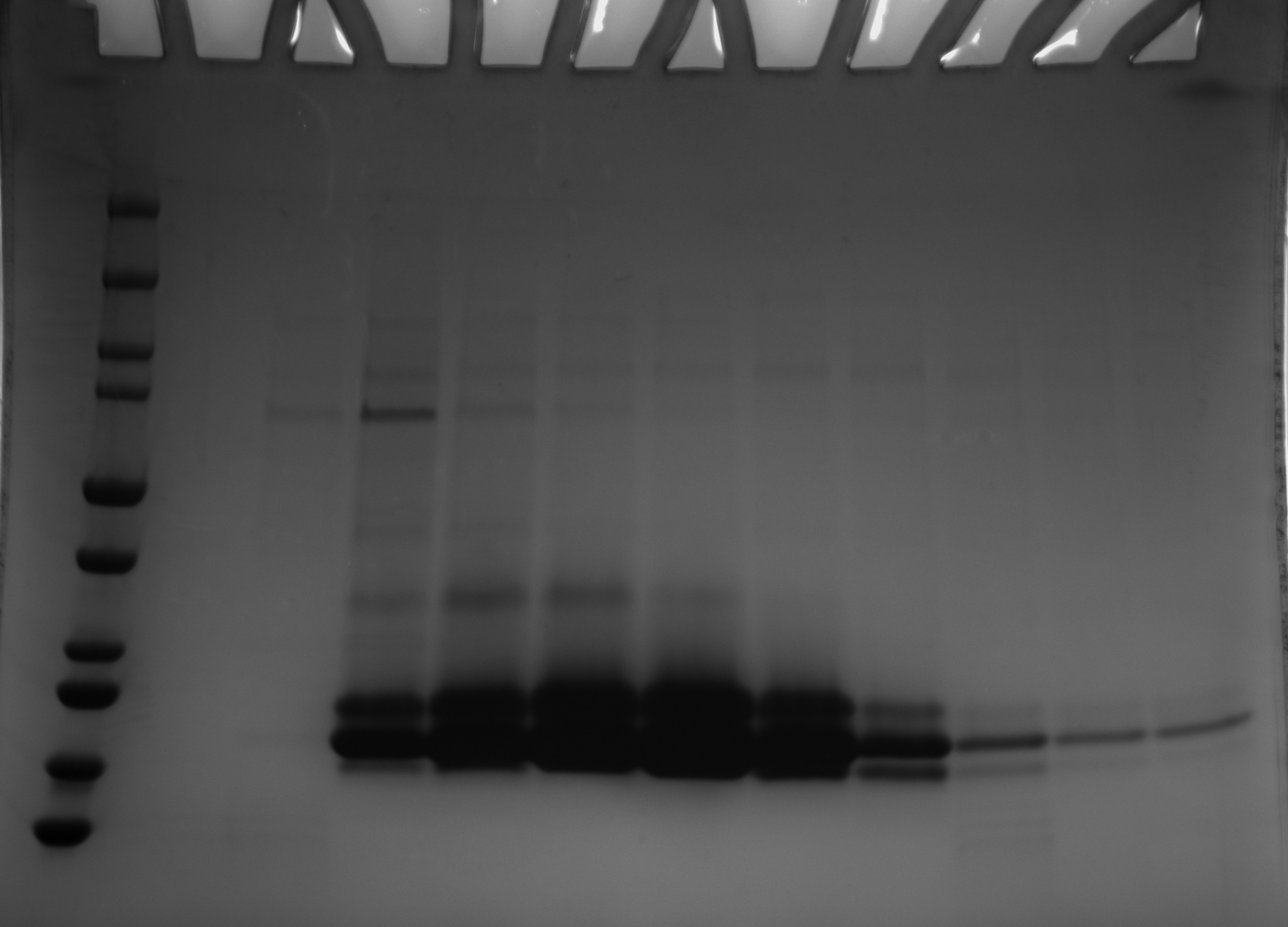

Supplement: Figure 3—figure supplement 1—source data 1. [file elife-76729-fig3-figsupp1-data1.zip › Figure3-figuresupplement1-Source Data/20180920-hSPRR1B-SD75.tif]

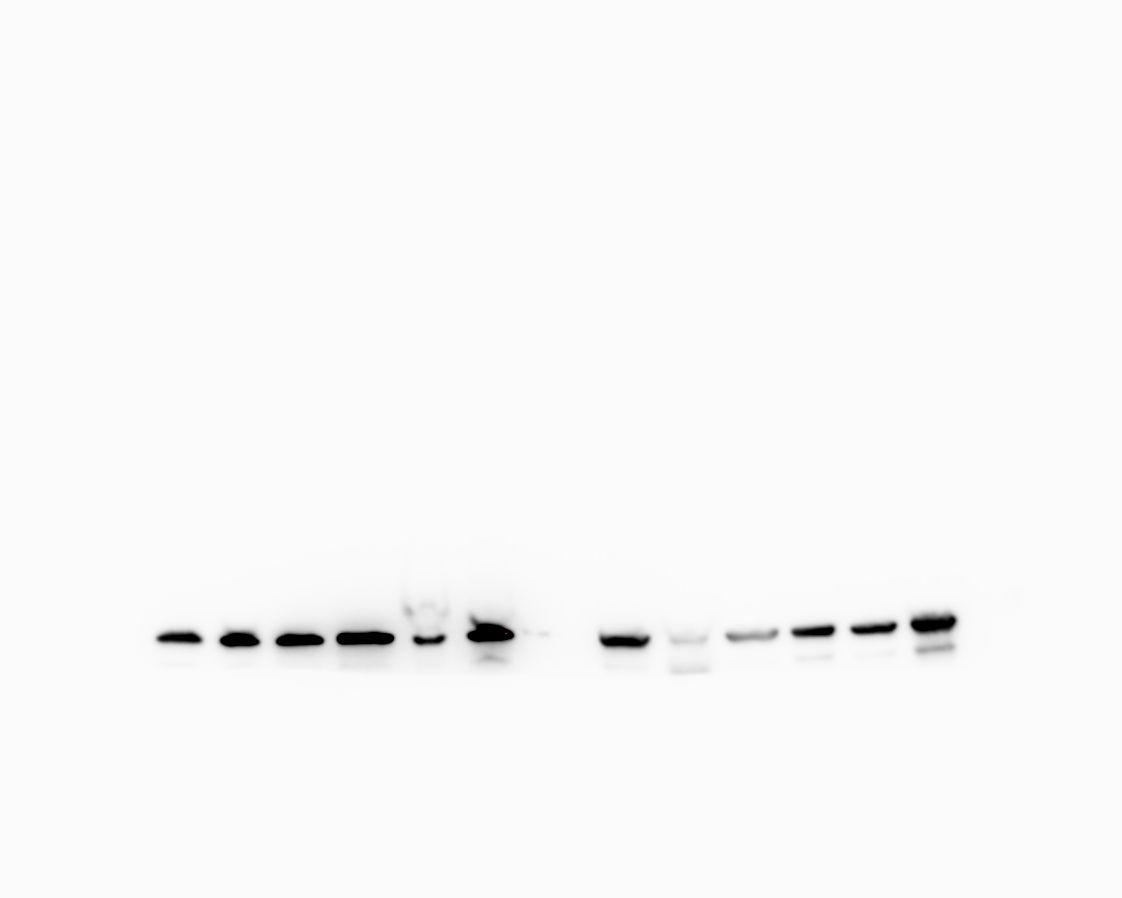

Supplement: Figure 4—figure supplement 1—source data 1. [file elife-76729-fig4-figsupp1-data1.zip › Figure 4_figuresupplement 1-Source Data/GAPDH(chemiluminescence).jpg]

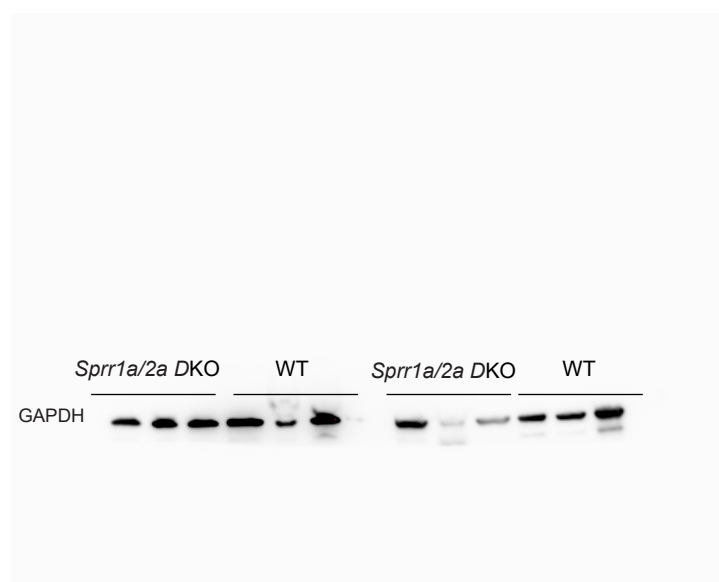

Supplement: Figure 4—figure supplement 1—source data 1. [file elife-76729-fig4-figsupp1-data1.zip › Figure 4_figuresupplement 1-Source Data/GAPDH(chemiluminescence).pdf]

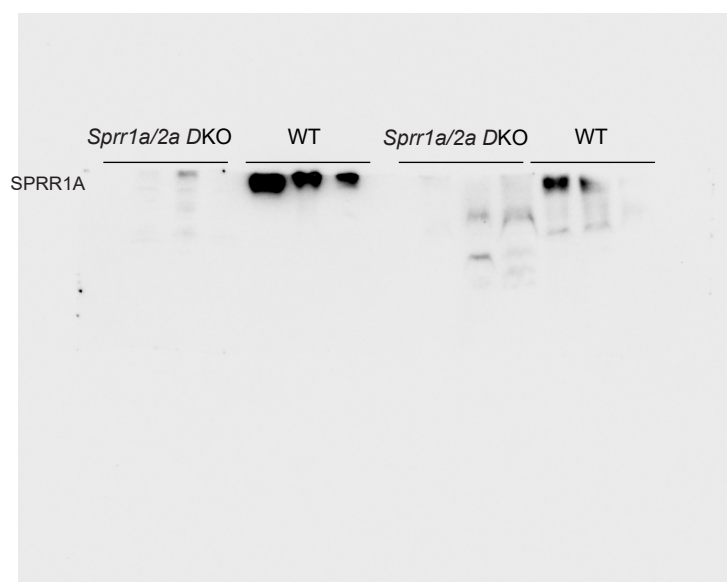

Supplement: Figure 4—figure supplement 1—source data 1. [file elife-76729-fig4-figsupp1-data1.zip › Figure 4_figuresupplement 1-Source Data/SPPR1A(Chemiluminescence).pdf]

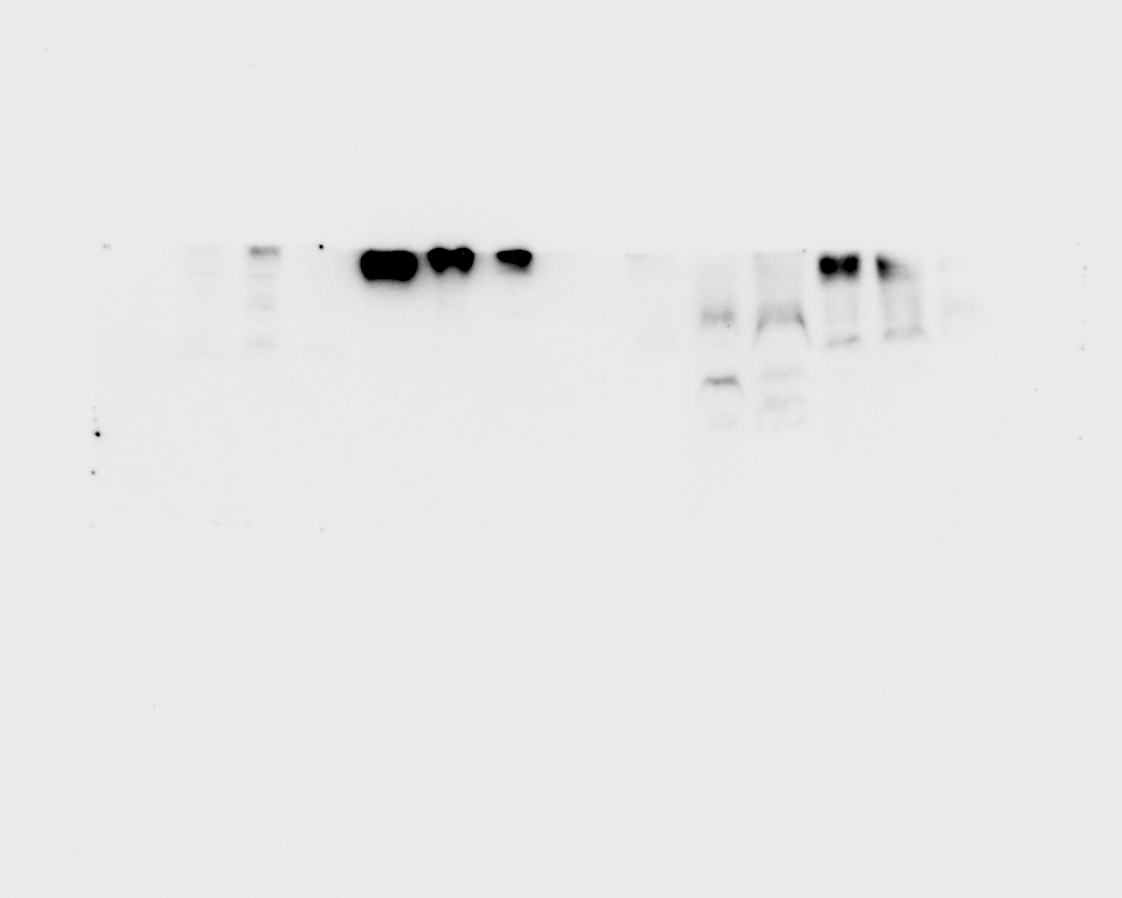

Supplement: Figure 4—figure supplement 1—source data 1. [file elife-76729-fig4-figsupp1-data1.zip › Figure 4_figuresupplement 1-Source Data/SPRR1a(Chemiluminescence).jpg]
